# Shift of bacterial communities in heavy metal-contaminated agricultural land during a remediation process

**DOI:** 10.1371/journal.pone.0255137

**Published:** 2021-07-23

**Authors:** Chi-Chun Huang, Chih-Ming Liang, Ting-I Yang, Jiann-Long Chen, Wei-Kuang Wang

**Affiliations:** 1 Endemic Species Research Institute, Jiji, Nantou, Taiwan; 2 Department of Environmental Engineering and Science, Feng Chia University, Taichung, Taiwan; Pingtung University of Science and Technology, TAIWAN

## Abstract

Anthropogenic activities accompanied by heavy metal waste threaten the environment. Heavy metal pollution alters the soil microbial community composition, and the microorganisms that adapt to this stress increase in abundance. The remediation process of contaminated soil not only reduces the concentration of heavy metals but also alters the bacterial communities. High-throughput 16S rDNA sequencing techniques were applied to understand the changes in soil microbial communities. Using the remediation approach of the soil mixing, the concentrations of heavy metals in the contaminated areas were diluted and the soil environment was changed. The change of soil environment as a disturbance contributed to the alteration of microbial diversity of the remediated areas. The pH and heavy metals (Cr, Cu, Ni, and Zn) were the most influential factors driving the changes in community structure. The bacterial community structure was significantly different among sample areas. The decrease of heavy metals in soil may be the important factors that changed the microbial composition. This study provides the better understanding of the changes in composition of microbial communities affected by the remediation process in heavy metal-contaminated soil.

## Introduction

The rapid development of civilization poses a great threat to the environment. Wastewater irrigation, sludge applications, solid waste disposal, automobile exhaust and industrial waste dumping lead to highly contaminated areas worldwide. The presence of environmental pollution may be lethal or toxic to living things in contaminated areas [1]. When areas are contaminated with heavy metals, soil microorganisms are highly sensitive to the impacts of heavy metals [2, 3]. The number of soil microorganisms decreases by direct killing or biochemical deactivation [4, 5]. Soil microorganisms, which are integral components in ecosystems, play important roles in cycling nutrients, maintaining soil structure, regulating plant growth, and combating harmful pathogens [6]. Heavy metal pollution will alter the soil microbial community composition, and the microorganisms that can adapt to these stress increase in abundance [7]. The change in soil microbial community composition that may affect the ability of organic degradation causes the loss of soil fertility [8].

In Taiwan, rapid industrial development has resulted in soil pollution on farmland [9, 10] and terrestrial biota [11]. The industrial wastewater with heavy metals was discharged directly into the farmland irrigation in past decades [12, 13]. Irrigation become a significant source of heavy metal-contaminated farmland, especially when the water used comes from rivers that have received high pollutant loads. A serious agricultural issue appeared that about 2.6% farmland were contaminated by heavy metals according to Environmental Protection Administration Executive Yuan, R. O. C. (Taiwan) (hereinafter referred to as the Taiwan EPA, https://www.epa.gov.tw/ENG/). According to Taiwan EPA, the main heavy metal pollutants in soil include arsenic (As), cadmium (Cd), chromium (Cr), copper (Cu), lead (Pb), zinc (Zn), mercury (Hg), and nickel (Ni). Cu and Zn are essential elements for plant growth and are referred to as micronutrients. At high concentrations, however, these elements are toxic to plants. As, Cd, Cr, Pb, Hg, and Ni have toxic effects on living organisms [14]. Most heavy metals in soil can accumulate in crops and can be transferred to other media through the food chain. Crops polluted by heavy metals could be harmful to humans Food crops may be contaminated by heavy metals and accumulate high concentrations of heavy metals. By the biological magnification effect, these crops have threatened human health [15, 16]. The ingestion of contaminated crops causes serious human health issues, such as gastrointestinal cancer, fragile immunological mechanisms, mental growth retardation, and malnutrition. Through dietary intake, the accumulation of heavy metals in the human body also leads to the depletion in essential nutrients and reduction in immunological defenses [17].

A number of studies have shown that heavy metals impact soil bacteria at varying degrees. Long-term exposure to heavy metals (Cu, Ni and Zn) could alter the microbial structure of soil [7]. Xiao et al. [12] revealed that the bacterial community structure is mainly altered by soil organic matter, Cr and pH. The diversity and species richness were decreased by Zn, and the core microbiome of contaminated soil contained *Holospora* and *Sphingomonas* [13]. The study of Li et al. [14] showed that bacterial responses to heavy metals vary. Some bacteria, e.g., *Acidobacteria_Gp* and *Proteobacteria_thiobacillus*, were positively correlated with Cd, while other bacteria, e.g., *Longilinea*, *Gp2* and *Gp4*, were negatively correlated with Cd. The effects of heavy metals on microbes may be different at varying concentrations. A high concentration of Hg caused severe losses of diversity and shifted the microbial community structures, whereas a low concentration of Hg increased microbial diversity [17, 18]. The soil mixing is an application that removes contaminants from source media. The contaminated soil was mixed with uncontaminated soil to reduce the concentrations of heavy metals in the soil. This application aimed to dilute the concentration of heavy metals in soil and allow the metals to degrade naturally. Although numerous studies have examined the changes in microbial compositions under the effects of heavy metals [13, 14, 19], few studies have focused on the changes in microbes under artificial remediation. However, traditional microbiological methods based on culture-based techniques and molecular fingerprinting often underestimates the number and diversity of soil microorganisms. Understanding the change in the diversity of soil microbial populations in contained soil will help clarify the ecological role of soil microorganisms. High-throughput sequencing techniques such as Illumina sequencing of 16S rDNA amplicons provide not only higher resolution approaches for studying the phylogenetic composition of microbial communities [16] but also a more detailed understanding of the components of soil microbial communities in heavy metal-contaminated environments [14]. The application of this method may provide better insight into the structure of soil microbial communities that were influenced by heavy metals. The study aimed to elucidate the following questions: First, what are the bacterial diversity after soil remediation? Second, are the bacterial compositions different after soil remediation? Third, are the changes of heavy metals concentrations associated with the compositions of soil microorganisms? Finally, are there any microbes associated with the heavy metals? If yes, what are their roles?

## Materials and methods

### Study areas

The study was conducted in Nantou (23°54’ N, 120°41’ E) and Changhua (24°05’ N, 120°31’ E) counties in central Taiwan. The web sites of soil and groundwater pollution remediation funds present the information about the Taiwan soil and groundwater pollution (https://sgw.epa.gov.tw/). In Nantou County, the mean annual temperature is 22°C, with a mean rainfall of 2400 mm. The elevation of the sample areas were about 200 m. The sample areas were described as As- and Pb-contaminated areas since 2002. In Changhua County, the mean annual temperature is 24°C, with a mean rainfall of 1300 mm. The elevation of the sample areas were about 20 m. Because of the high concentrations of Cr, Cu, Ni, and Zn, the sample areas were described as contaminated areas in 2013. The industrial plants near the sample areas may be the main sources of pollution. These farmlands were contaminated via irrigation water. The agricultural activities were prohibited since these farmlands were described as heavy-metals contaminated areas, these idle farmlands were overgrown with weeds.

### Sample collection and environmental factors

Three samples of the polluted areas of Nantou (PN) and Changhua (PC) were collected in October 2017, respectively. To dilute the heavy metal concentrations in soil, the soil mixing was applied to remediate the sample areas during December 2017 to Jane 2018. An excavator was used to remove 0–30 cm of the surface soil and plow the maximum turning depth of 40 cm. Three replicated samples from the remediated areas of Nantou (RN) and Changhua (RC) were further collected in December 2018. The soil samples were collected under the license with permission granted by the Environmental Protection Bureau of Nantou County Government (permit number 1070025486) and Changhua County Government (permit number 1060114612), respectively. The mean values of temperature (Temp) and precipitation (Precp), which ranged from September 2016 to October 2017 for polluted areas and from November 2017 to December 2018 for remediated areas, were collected from the website of the central weather bureau (https://www.cwb.gov.tw/V8/C/). The ICP-AES was used to analyze heavy metal elements in the soil samples collected at depths of 10–20 cm as described by Tao et al. [20].

### DNA extraction and 16S rDNA gene amplification

DNA was also extracted from the same soil samples which were collected for heavy metal analyzes. The total genomic DNA from the soil samples was extracted using the DNeasy PowerSoil Kit (Qiagen). For each sample, the DNA quality was evaluated by a Nanodrop Spectrophotometer (ND-1000) and agarose gel electrophoresis. For metagenomic analysis, 10 ng of DNA was used to amplify the 16S rDNA gene, and two universal microbial primers, 341 F (CCTACGGGNGGCWGCAG) and 805R (GACTACHVGGGTATCTAATCC), were used to target the variable V3-V4 region [21]. The sequencing libraries were generated using the TruSeq nano-DNA Library Prep Kit (Illumina, USA) following the manufacturer’s recommendations, and index codes were added. The prepared library quality was quantified on a Qubit@ 2.0 Fluorometer (Thermo Scientific) and validated by an instrument (Bioanalyzer 2100, Agilent). The library was sequenced on an Illumina MiSeq platform with paired-end reads (2 x 300 bp); image analysis and base calling were conducted by Illumina MiSeq Control Software (MCS).16S rDNA sequences have been submitted to Sequence Read Archive (SRA) at NCBI metagenome resources. The following accession numbers were BioProject PRJNA681435.

### Analyses of microbial community composition and diversity

The sequences were preprocessed with the FastQC tool (Andrews et al. 2015). The remaining sequence data were then processed using two software programs, mothur 1.39.5 [22] and QIIME 1.9.0 [23], according to the mothur SOP. In QIIME, forward and reverse reads were joined with join_paired_ends.py. Chimeras were identified and filtered with the VSEARCH algorithm [24]. Finally, the tool was used to pick closed-reference OTUs from the SILVA database (SILVA version 132) [25] and representative sequences with 99% similarity. The pipeline for mothur was also started by joining the forward and reverse reads. Sequences were then preclustered and classified using a Bayesian classifier **(**Classify.seqs**)** with the same cut-off for sequence identity and reference databases. The OTUs were summarized by a mothur-formatted training set provided by the Schloss lab based on the Ribosomal Database Project [26] reference taxonomy. The results are deposited into the NCBI sequence read archive (SRA) database under accession number: SAMN16953900 –SAMN16953911.

### Statistical analyses

Rarefaction, alpha diversity (Chao1, Shannon, and Simpson diversity indexes), nonmetric multidimensional scaling (NMDS) and heatmap figures were generated in Vegan packages in R (http://www.r-project.org). To investigate the relationships between soil bacterial community composition and environmental factors, the OTUs from all soil samples were analyzed by redundancy analysis (RDA) using Vegan packages in R 4.0.2. Differences in the environmental factors of sample areas were tested by one-way analysis of variance (ANOVA). P < 0.05 was considered significant. Differences in community structure were tested using PERMANOVA, which was performed using adonis with 999 permutations from Vegan packages in R 4.0.2 [27].

## Results

### Environmental factors in the soil samples

The Temp, Precp and soil pH from the sample areas presented in Table 1. The mean Temp, Precp and pH were 24.9, 23.0, 23.5, and 21.7°C, 191.9, 123.8, 119.2, and 91.0 mm, and 6.0, 7.4, 5.3, and 6.3 in PN, PC, RN, and RC, respectively. Higher values of Temp, Precp and pH were detected in PN and PC compared to RN and RC, respectively.

**Table 1:** The environmental factors of soil samples.

	Temp (°C)	Precp (mm)	pH	As (mgKg-1)	Cd (mgKg-1)	Cr (mgKg-1)	Cu (mgKg-1)	Ni (mgKg-1)	Pb (mgKg-1)	Zn (mgKg-1)
PN	24.9 ± 4.2	191.9 ± 300.0	6.0	253.0 ± 146.1	4.3 ± 3.7	31.3 ± 5.0	82.7 ± 32.7	34.0 ± 6.4	2048.0 ± 1571	105.7 ± 8.4
PC	23.0 ± 4.3	123.8 ± 187.6	7.4	66.3 ± 0.5	11.7 ± 15.8	307.7 ± 169.0	330.0 ± 77.1	378.7 ± 206.6	37.7 ± 5.9	499.3 ± 264
RN	23.5 ± 4.3	119.2 ± 154.1	5.3	150.3 ± 46.3	12.7 ± 13.9	24.7 ± 8.3	0.0 ± 0.0	27.0 ± 5.4	945.3 ± 498.6	91.7 ± 5.3
RC	21.7 ± 4.5	91.0 ± 145.52	6.3	64.0 ± 0.8	4.0 ± 2.8	34.3 ± 8.7	8.0 ± 11.3	60.3 ± 4.8	12.7 ± 5.4	107.7 ± 12.5

Data are the means ± standard deviation. Temp, annual mean temperature; Precp, annual mean precipitation. PN, Nantou polluted area; PC, Changhua polluted area; RN, Nantou remediated area; RC, Changhua remediated area.

The concentrations of heavy metals in the soil samples collected from the sample areas are presented in Table 1. According to the farm soil pollution control standards of Taiwan (As, 60 mg kg^-1^; Cd, 5 mg kg^-1^; Cr, 250 mg kg^-1^; Cu, 200 mg kg^-1^; Ni, 200 mg kg^-1^; Pb, 500 mg kg^-1^; and Zn, 600 mg kg^-1^), the average concentrations of heavy metals except for Zn in the sample areas exceeded those in the control standards. Excess concentrations of As (253.0 ± 146.1 mg kg^-1^) and Pb (2048.0 ± 1571.9 mg kg^-1^) were detected in PN. Excess concentrations of As (66.3 ± 0.5 mg kg^-1^), Cd (11.7 ± 15.8 mg kg^-1^), Cr (330.0 ± 77.1 mg kg^-1^), Cu (307.7 ± 169.0 mg kg^-1^) and Ni (378.7 ± 206.6 mg kg^-1^) were detected in PC. Excess concentrations of As (150.3 ± 46.3 mg kg^-1^), Cd (12.7 ± 13.9 mg kg^-1^), and Pb (945.3 ± 498.6 mg kg^-1^) were detected in RN. Excess concentrations of As (64.0 ± 0.8 mg kg^-1^) was detected in RC. The concentrations of Cu, Cr, Ni, and Zn were highest in PC, whereas the concentrations of As and Pb were highest in PN. Except for Cd, the average concentrations of the other heavy metals were lower in remediated samples than in polluted samples. The concentrations of As, Cr, Cu, Ni, Pb and Zn in RC were significantly different (P < 0.05) compared to PC. The concentration of Cr in RN was significantly different (P < 0.05) compared to PN.

### Soil microbial community composition and diversity

**Fig 1:**
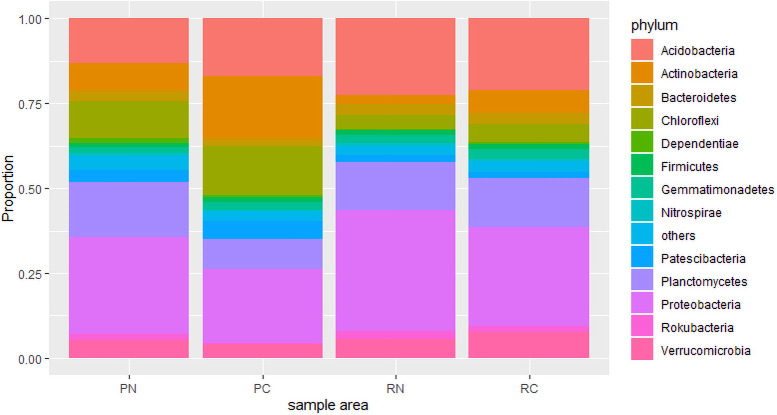
Relative abundance (%) of the dominant bacteria at the phylum level. PN, Nantou polluted area; PC, Changhua polluted area; RN, Nantou remediated area; RC, Changhua remediated area. The taxa of low abundance are pooled as “others”.

The 16S rDNA sequencing analyses generated 677,680 raw reads, with 622,970 quality reads across all analyzed samples. The number of quality reads per sample ranged from 40,045 to 69,749, with an average of 56,473. In total, 55,824 OTUs were identified. The shape of the rarefaction analysis tended to approach the saturation plateau, indicating the sequencing depths were reasonable for further analysis (S1 Fig). The soil microbial communities were predominantly composed of bacteria, which accounted for 99.4% of the classifiable 16S rDNA sequences, whereas only a low proportion of the sequences (0.5%) were assigned to archaea. Proteobacteria (21.7%–35.6%), Acidobacteria (13.2%–22.4%), and Planctomycetes (8.9%–16.1%) dominated the classified bacterial phyla and accounted for approximately 28.9%, 18.4%, and 13.4% of all the sequences, respectively. The relative abundance of each bacterial phylum varied among the different areas (Fig 1).

Proteobacteria, Acidobacteria and Planctomycetes were the dominant phyla in the sample areas. Six phyla (11.8%) were only detected in the polluted areas, while 1 phylum (1.9%) was only detected in the remediated areas (S2A Fig). One way ANOVA was applied to test the differences between polluted and remediated areas in Changhua and Nantou, respectively. In Changhua, the relative abundances of OTUs of Actinobacteria (18.4% in PC, 6.7% in RC), Chloroflexi (14.2% in PC, 5.2% in RC) and Patescibacteria (5.4% in PC, 1.8% in RC) were higher in PC and significantly different from those in RC (P < 0.05), and those of Planctomycetes (8.9% in PC, 14.4% in RC), Proteobacteria (21.9% in PC, 29.2% in RC) and Verrucomicrobia (4.0% in PC, 7.7% in RC) were higher in RC and significantly different from those in PC (P < 0.05). In Nantou, the relative abundances of OTUs of Chlamydiae (1.0% in PN, 0.2% in RN), Chloroflexi (10.9% in PN, 4.1% in RN), and Dependentiae (1.6% in PN, 0.5% in RN) were higher in PN and significantly different from those in RN (P < 0.05), and those of Acidobacteria (13.2% in PN, 22.4% in RN) was higher in RN and significantly different from those in PN (P < 0.05).

**Fig 2:**
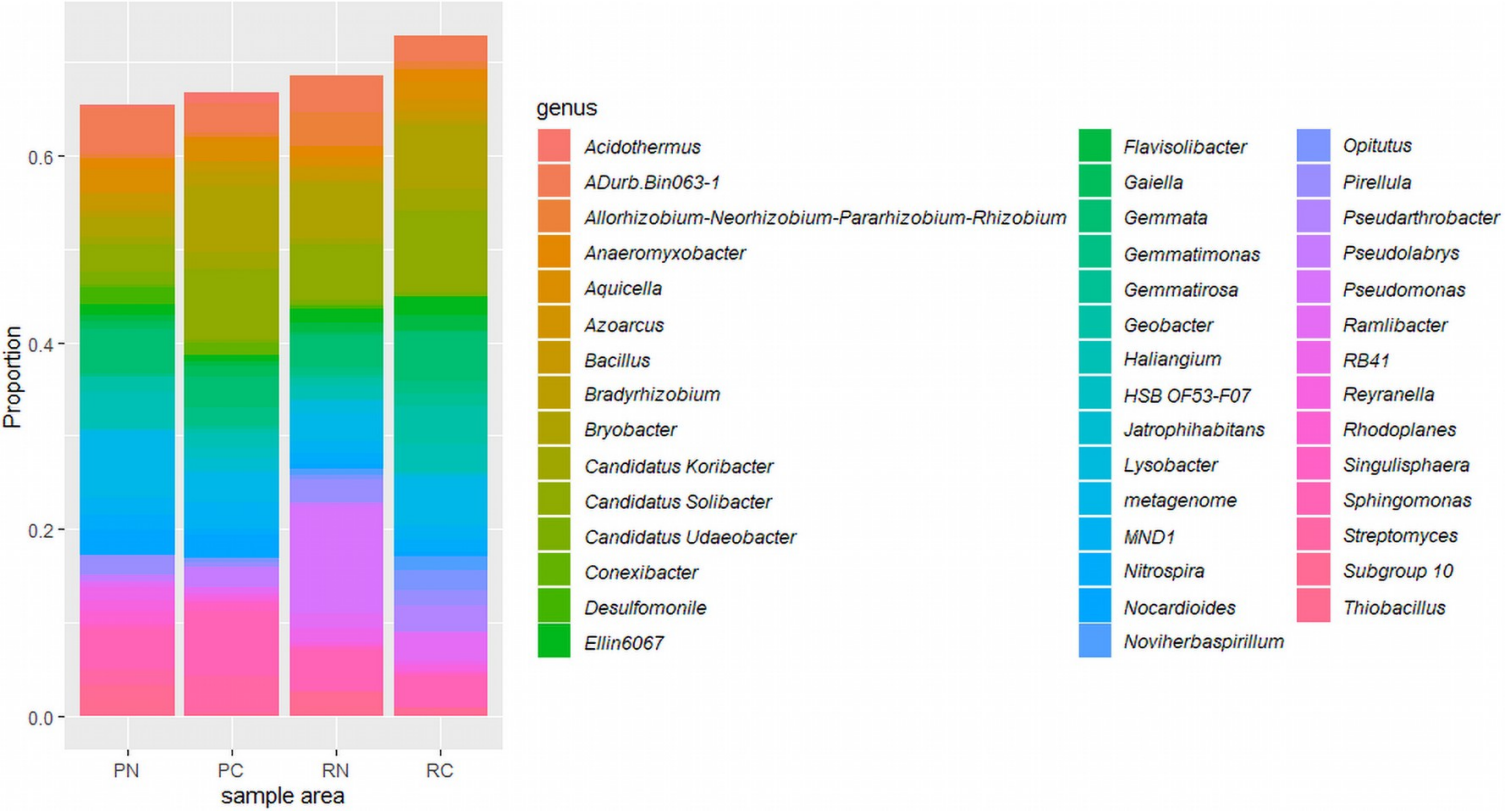
Relative abundance (>1%) of the dominant bacteria at the genus level. PN, Nantou polluted area; PC, Changhua polluted area; RN, Nantou remediated area; RC, Changhua remediated area.

At the genus level, an average abundance of > 1% was defined as dominant except for the unknown genus (Fig 2). 548 genera (33.6%) were only detected in the polluted areas, and 312 genera (22.4%) were only detected in the remediated areas (S2B Fig). One way ANOVA was applied to test the differences between polluted and remediated areas in Changhua and Nantou, respectively. In Changhua, the relative abundance of OTUs of *Conexibacter* (1.2% in PC, 0.1% in RC) was higher in PC and significantly different from those in RC (P < 0.05), and those of *Ellin6067* (0.7% in PC, 2.0% in RC), *Noviherbaspirillum* (0.1% in PC, 1.6% in RC) and *Ramlibacter* (0.1% in PC, 2.9% in RC) were higher in RC and significantly different from those in PC (P < 0.05). In Nantou, the relative abundance of OTUs of *Haliangium* (4.0% in PN, 1.6% in RN) was higher in PN and significantly different from those in RN (P < 0.05), and those of *Lysobacter* (0.2% in PN, 1.3% in RN) and *Ramlibacter* (0.4% in PN, 1.7% in RN) was higher in RN and significantly different from those in PN (P < 0.05).

The alpha diversity of the 16S rDNA sequencing results were used to evaluate the microbial richness and diversity (Table 2). The OTUs ranged from 1314–1846. The chao1, shannon and simpson indices ranged from 782–1006, 5.494–5.811, and 0.982–0.992, respectively. Higher OTUs and alpha diversity indices in PN were higher than those in RN. Higher OTUs and Chao1 and lower Shannon and Simpson indices were detected in PC compared to RC. However, no significant differences in bacterial diversity and abundance were detected among the sample areas (PN, PC, RN, and RC).

**Table 2:** 16S rDNA sequencing results and diversity estimates for each sampling site.

	Sequence results		Diversity estimates		
	Sequences	OTUs	Chao1	Shannon	Simpson
PN	174045	1846	1006 ± 92	5.811 ± 0.169	0.992 ± 0.002
PC	155502	1416	782 ± 31	5.494 ± 0.095	0.990 ± 0.002
RN	159532	1634	896 ± 219	5.500 ± 0.337	0.982 ± 0.011
RC	130288	1314	727 ± 42	5.591 ± 0.029	0.992 ± 0

PN, Nantou polluted area; PC, Changhua polluted area; RN, Nantou remediated area; RC, Changhua remediated area.

### Relative influences of physical properties and heavy metals on microbial compositions

**Fig 3:**
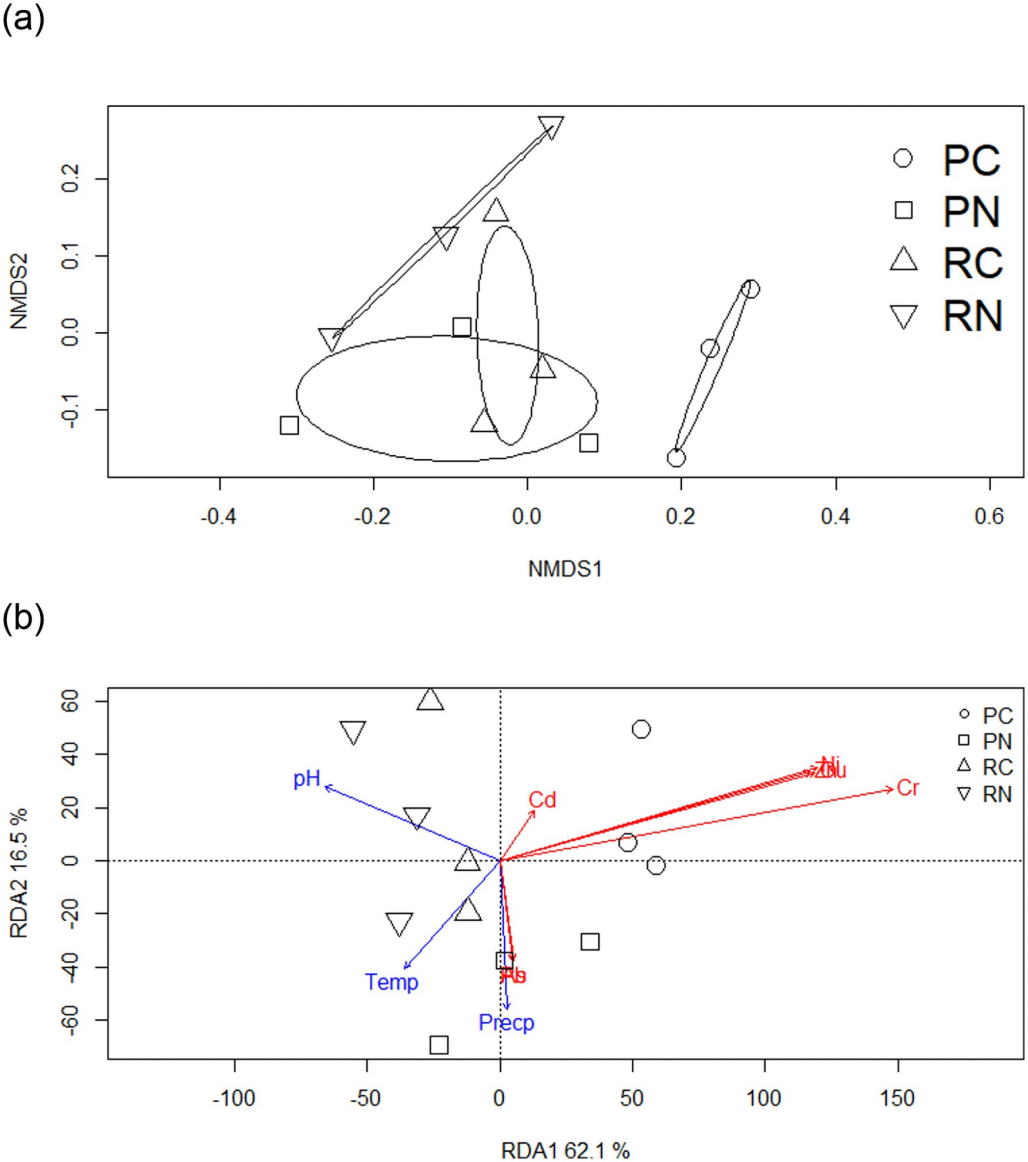
(A) Nonmetric multidimensional scaling (NMDS) ordination of the soil samples and (B) redundancy analysis (RDA) show the correlation among bacterial community and a subset of seven heavy metals at the phylum level. PN, Nantou polluted area; PC, Changhua polluted area; RN, Nantou remediated area; RC, Changhua remediated area.

At the phylum level, the ADONIS results indicated that the significantly differences of microbial community composition were detected among the sample areas (PN, PC, RN, and RC). The nonmetric multidimensional scaling (NMDS) ordination (Fig 3A) was performed that PC was separated from the other areas. The RDA analyses showed that the environmental factors explained 78.5% (RDA1 explained 62.1% and RDA2 explained 16.5%) of the variances of bacterial community structures (Fig 3B). The soil pH (RDA1 = -92.0%, r2 = 0.70, P = 0.007) significantly negatively correlated with RDA1 and the Cu (RDA1 = 96.1%, r2 = 0.55, P = 0.002), Cr (RDA1 = 98.3%, r2 = 0.82, P = 0.005), Ni (RDA1 = 95.7%, r2 = 0.56, P = 0.01), and Zn (RDA1 = 96.0%, r2 = 0.54, P = 0.01) significantly positively correlated with RDA1, indicating that these factors were critical for explaining the variations in the bacterial community structure. A Mantel test was performed to analyze the correlation between soil microbial community structure and environmental factors. The Mantel test results showed that significant correlation was detected between soil bacteria and pH (mantel r = 0.44, P = 0.004). Soil bacteria were significantly correlated with Cu (mantel r = 0.275, P = 0.01), Cr (mantel r = 0.663, P = 0.01), Ni (mantel r = 0.414, P = 0.01) and Zn (mantel r = 0.509, P = 0.001).

At OTU level, the ADONIS results indicated that the microbial community composition was significantly different among the sample areas (PN, PC, RN, and RC). The soil pH (RDA1 = -78.5%, r2 = 0.60, P = 0.07) was significantly negatively correlated with RDA1 and the Cu (RDA1 = 31.6%, r2 = 0.74, P = 0.01), Cr (RDA1 = 66.7%, r2 = 0.41, P = 0.05), Ni (RDA1 = 40.8%, r2 = 0.85, P = 0.001), and Zn (RDA1 = 37.4%, r2 = 0.72, P = 0.004) significantly positively correlated with RDA2. The Mantel test results showed that significant correlation was detected between soil bacteria pH (mantel r = 0.417, P = 0.005). Soil bacteria were significantly correlated with Cu (mantel r = 0.395, P = 0.02), Cr (mantel r = 0.575, P = 0.02), Ni (mantel r = 0.583, P = 0.01) and Zn (mantel r = 0.427, P = 0.01).

**Fig 4:**
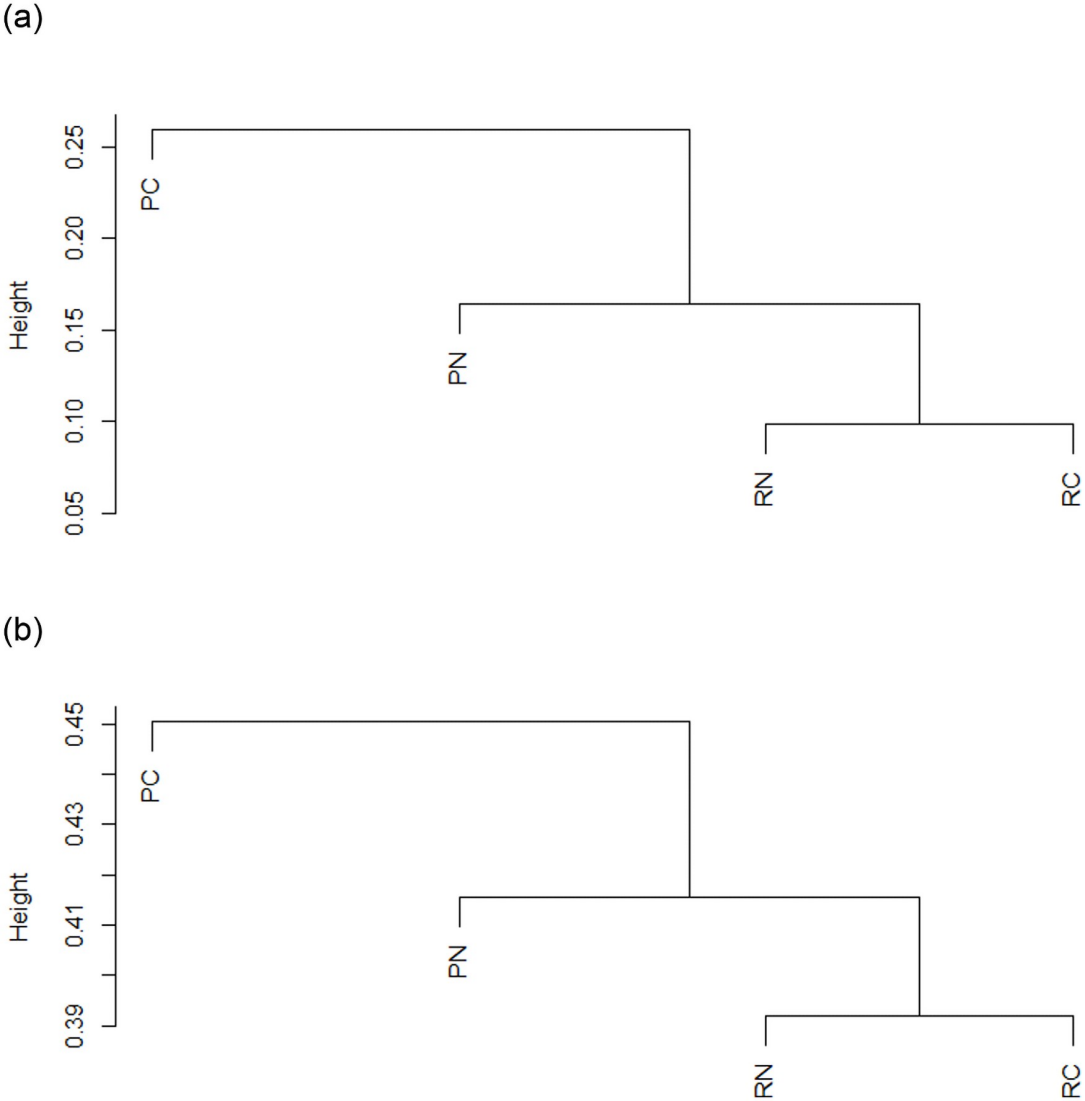
The dendrogram using the unweighted pair group method with arithmetic mean (UPGMA) method at (A) phylum level and (B) genus level. PN, Nantou polluted area; PC, Changhua polluted area; RN, Nantou remediated area; RC, Changhua remediated area.

To determine the degree of similarity among the samples, UPGMA was performed based on Bray-Curtis distances at phylum (Fig 4A) and genus levels (Fig 4B). The UPGMA results showed the similar patterns that PC was distantly related with the other areas, while the RC and RN were closely related.

**Fig 5:**
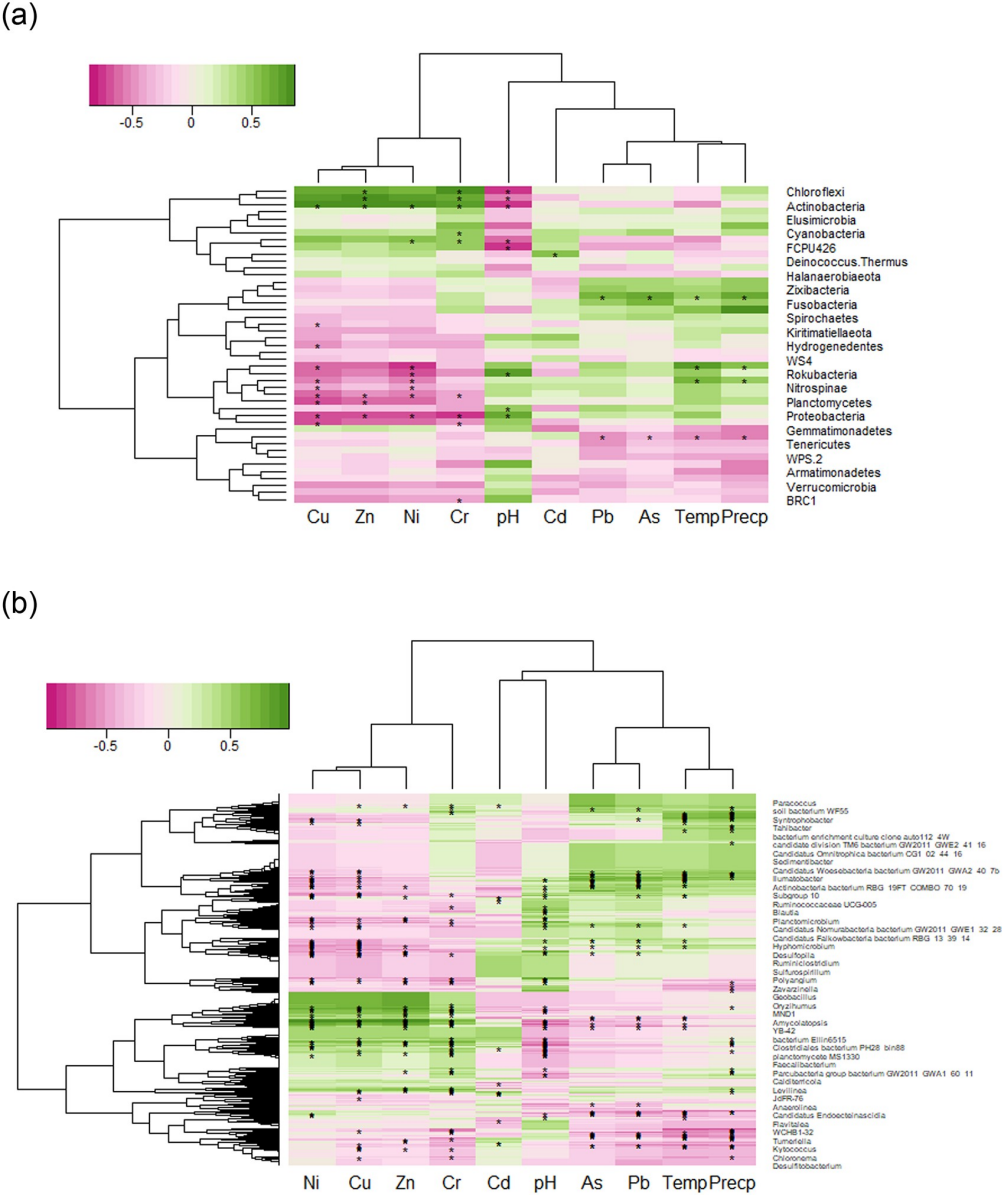
Spearman correlation analyses show that the bacterial phyla are significantly positively/negatively correlated with physical properties and heavy metals at (A) phylum level and (B) genus level. The x-axis and y-axis of the heatmap are environmental factors and phyla/genera, respectively. The r-value is shown in different colors in the graph. The values marked * indicate a significance test at P < 0.05.

Spearman correlation analysis revealed that the soil communities have different response to the environmental factors (Fig 5). This analysis divided the properties into two groups at phylum and genus levels. One group contains Cd, Cr, Cu, Ni, and Zn, the other group contains As, Pb, Precp, pH and Temp. Two clusters were detected at phylum and genus levels, respectively. At phylum levels, cluster 1 was significantly positively related with Cr, Cu, Ni, and Zn. Cluster 2 was significantly negatively related with Cr, Cu, Ni, and Zn. Similar pattern was detected at genus level. Cluster 1 was significantly positively related with Cr, Cu, Ni, and Zn, while cluster 2 was significantly negatively related with Cr, Cu, Ni, and Zn.

## Discussion

### Sequencing diversity of sample areas

Human activity may lead to variations in microbial composition and diversity [19]. Hong, et al. [20] showed that the diversity of polluted areas was significantly higher than that of unpolluted areas. However, the other authors have shown that heavy metal polluted areas had severely less diversity, and the bacterial and fungal community structure and composition were shifted [28, 29]. The microbial community may potentially survive in polluted areas by tolerating pollutants by detoxification mechanisms [30, 31]. In this study, PC and PN were contaminated by heavy metals for approximately 4 and 15 years, respectively. After remediation by soil mixing, the change of alpha diversity (Table 2) suggested that soil mixing as a disturbance affect not only the concentrations of heavy metals but also the alpha diversities of microorganisms. Berga et al., [3] found that disturbances that cause changes in resources or in the physical environment influenced the bacterial community structure. Ager et al., [32] showed that the anthropogenic disturbance affect the bacterial community structure and it took longer time to recover pre-disturbance level under stronger intensity of disturbance. The environmental changes, such as acidification and wildfire, can cause a sudden disturbance that results in the loss of diversity [33]. The change of environmental resource can lead to the change in biodiversity [34]. Human disturbance may result in short-term unstable communities, as revealed by reduction of soil microbial diversity and shift of composition. The remediated processes of soil mixing were composed of three steps, the removal of contaminated soil, the exchange of surface and deeper soil, and the addition of improved soil. Hence, the remediated processes substantially decreased the concentrations of heavy metals and changed the soil environment (Table 1). The change of soil environment as a disturbance may contribute to the alteration of microbial diversity of the remediated areas.

### Taxonomy composition of sample areas

The most dominant phyla of soil microorganisms across the globe are Proteobacteria, Acidobacteria, Actinobacteria and Planctomycetes [35, 36]. The results of this study revealed that Proteobacteria, Acidobacteria, Planctomycetes, Actinobacteria and Chloroflexi were the top five dominant phyla (76.7%–80.5%) in the sample areas (Fig 1). The disturbance of remediation and dilution of heavy metals have resulted in the changes the soil environment and the microorganism compositions. For the phyla with relative abundance > 1%, four phyla and six phyla in Nantou and Changhua were significantly different, respectively. Wu et al., [37] revealed that proportion of the Proteobacteria and Actinobacteria were remarkably higher in revegetated tailings, while Chloroflexi performed reversely. The change of relative abundance indicated that each bacterial phylum responded differently after the soil remediation. Chloroflexi was the only phylum showing significantly decrease in both Nantou and Changhua. Proteobacteria and Acidobacteria were the phyla displaying significantly increase in Nantou and Changhua, respectively. Zeng et al., [38] suggested that heavy metals increase the abundance of Chloroflexi, whereas decreased the abundance of Proteobacteria and Acidobacteria. Azarbad et al., [39] and Spain, Krumholz, and Elshahed [40] reported that Chloroflexi can adapt to the heavy-metal polluted environments. The remediated process through the excavation the polluted soil and the mix with unpolluted soil decreased the concentrations of heavy metals in soil and disturbed the bacterial compositions, as shown by the shift of relative abundance of OTUs in bacteria.

### Correlation between environmental factors and community structure

Soil pH and heavy metals were the most important factors that affect the microbial compositions in soil [38, 41]. Xiao et al., [42] showed that the soil organic matter, Cr, and pH are the factors altering the bacterial community structure. Wu et al., [19] showed that available phosphorus, soil moisture, and mercury are the three major drivers affecting the microbial assemblages. Das et al., [43] showed that the bacterial composition of high As-contaminated soils differs significantly from that of low As-contaminated soils. Fatimawali et al. [44] showed that the high level of mercury in the soil reduced the richness and diversity of bacterial phyla. In other words, the change of environmental factors in soil inevitably affected the bacterial community structure. In this study, the concentrations of heavy metals in soil were decreased by the soil remediation. The ADONIS results also showed that the microbial compositions were significantly different among sample areas (PC, PN, RC, and RN). The differentiation may be attributable to three possible reasons. First, the Temp, Precp and soil pH may have critical roles in changing the microbial composition [45–47]. Tran et al., [47] showed that the abundance of Firmicutes were influenced by temperature and precipitation across seasons. In this study, the microbial compositions had no significant correlations with Temp and Precp, implying that the alteration of Temp and Precp were not related with the microbial compositions. In contrast, soil pH was significantly negatively correlated with the soil bacteria community structures (P < 0.05). This result was in accordance with previous studies [42, 48, 49] that indicate microbial communities were affected by soil pH. Second, the heavy metal may cause a shift in the bacteria composition [44, 50, 51]. Song et al., [52] suggested that the heavy metals (Cd, Cu and Zn) change the microbial biomass and the bacterial community. The concentrations of As, Cr, Cu, Ni, Pb, and Zn in RC and that of Cr in RN were significant different compared to PC and PN, respectively. Significant positive correlation were detected between heavy metals (Cr, Cu, Ni, and Zn) and microbial communities. Such result may indicate that the decrease of heavy metals drove changes occurring in the community structure. Third, the comprehensive effects of the alteration of soil pH and heavy metals changed the bacterial communities. The mobility of heavy metals were affected by soil pH, which could transform inactive forms of heavy metals into active forms [53, 54]. Violante et al., [55] showed that pH has a strong influence on the dynamics of metal ions which are more mobile at lower pH. This study showed that both soil pH and heavy metals affect the soil microbial communities. The bacterial phyla or genera that are significantly positively related to heavy metals are always significantly negatively related to pH, and vice versa, the bacterial phyla or genera that are significantly negatively related to heavy metals are always significantly positively related to pH (Fig 5). The change of pH may influence the dynamics of heavy metals, resulting in the differentiation of bacterial community. RN and RC with lower values of pH and heavy metals had significantly different bacterial communities compared to PN and PC, respectively. These results suggested that pH and heavy metals have comprehensive effects on the bacterial communities.

### Comparison of bacterial community structures among areas

At the phylum level, the ADONIS results showed that the bacterial communities among sample areas were significantly different (P < 0.05). The UPGMA tree revealed that the PC samples were separated from the other areas (Fig 4A). The PN samples were clustered with remediated sample areas (RC and RN). The NMDS results showed that the PC samples were separated from the other areas. The RC samples closely related with the PN and RN samples. Similar results were observed at the genus level (Fig 4B). Gołebiewski et al., [13] showed that the specific effects of heavy metals can be seen even at a lower level. The analysis at the phylum and genus level represented the same patterns of community structure. Li et al. [14] found that soil microbes adapt to long-term heavy metal pollution through changes in microbial community composition and structure rather than changes in their diversity and evenness. These results demonstrated that the microbial communities of the remediated sample areas had significant differences compared to those of the polluted sample areas. Furthermore, the RN and RC that are in geographic separation showed that the bacterial communities of remediated areas have similar compositions (Fig 4). Xiong et al. [56, 57] showed that spatial distance contributes more to bacterial community variation. In contrast, the geographically distant sites had similar community compositions, suggesting that the compositions of bacterial communities have similar trends after the soil remediation.

### Differences of the bacterial genera among areas

Taxonomic classification revealed the differences in microbial composition among sample areas at the genus level. On the basis of the relative abundance of the genera, the genera with an average abundance of >1% in at least one group were defined as dominant. The genera *Pseudomonas*, *Candidatus Solibacter* and *Bryobacter* were more abundant in RN than in PN. The genera *Candidatus Solibacter*, *Gemmata* and *Ramlibacter* were more prevalent in RC than in PC (Fig 2). The genus *Pseudomonas* is ubiquitous in soil ecosystems and capable of metabolizing a wide range of organic and inorganic compounds [1]. *Candidatus Solibacter* was the abundant genus in the uncontaminated site [58]. The abundance of *Bryobacter* was negatively correlated with concentrations of Cr [12]. One way ANOVA was applied to test the differences between polluted and remediated areas in Changhua and Nantou, respectively. The decrease of abundance of *Conexibacter* and the increase of abundance of *Ellin6067*, *Noviherbaspirillum* and *Ramlibacter* in RC were detected compared to PC. The decrease of abundance of *Haliangium* and the increase of abundance of *Lysobacter* and *Ramlibacter* in RC were detected compared to PC. The remediated areas had alteration in microbial compositions. Although little is known about the effects of the specific bacteria on soil remediation, our results may be coincided with previous studies [59–61]. Khudur et al., [59] showed that *Conexibacter* was present in most co-contaminated soil. Song et al., [60] showed that the proportions of *Haliangium* were elevated at Cd polluted site. Remenar et al., [61] showed that lower abundance of *Haliangium* and *Ramlibacter* was found in Ni-contaminated soil. The concentrations of heavy metals that were related to the microbial communities were significant decrease in the remediate areas (Cr, Cu, Ni, and Zn in RC; Cr in RN). In spite of the residual heavy metals in the remediated areas, the drastic decrease of heavy metals in soil may be the key factors that contributed to the change of microbial composition.

## Conclusions

The remediation of polluted soil not only reduced the concentration of heavy metals but also altered the bacterial community structures. By the remediation, the concentrations of heavy metals in the polluted areas were diluted and the soil environment were changed. The change of soil environment as a disturbance may contribute to the alteration of microbial diversity of the remediated areas. The soil pH and heavy metals (Cr, Cu, Ni, and Zn) were the most influential factors driving the changes in community structure in this study. The bacterial community structures were significantly different among sample areas. The compositions of bacterial communities showed similar trends after the soil remediation. This study provides the better understanding of the changes in composition of microbial communities affected by the remediation process in the soil with heavy metal contamination.

## Supporting information

S1 Fig Rarefaction curves based on the number of sequences and operational taxonomic units. PN, Nantou polluted area; PC, Changhua polluted area; RN, Nantou remediated area; RC, Changhua remediated area.
(TIFF)

S2 Fig Venn diagram at the (a) phylum level (b) genus level. PN, Nantou polluted area; PC, Changhua polluted area; RN, Nantou remediated area; RC, Changhua remediated area.
(TIF)
