# Supplementary figures and images for: Shift of bacterial communities in heavy metal-contaminated agricultural land during a remediation process
Source: PLoS One. 2021 Jul 23;16(7):e0255137. doi: 10.1371/journal.pone.0255137 (PMC8301633; doi:10.1371/journal.pone.0255137)

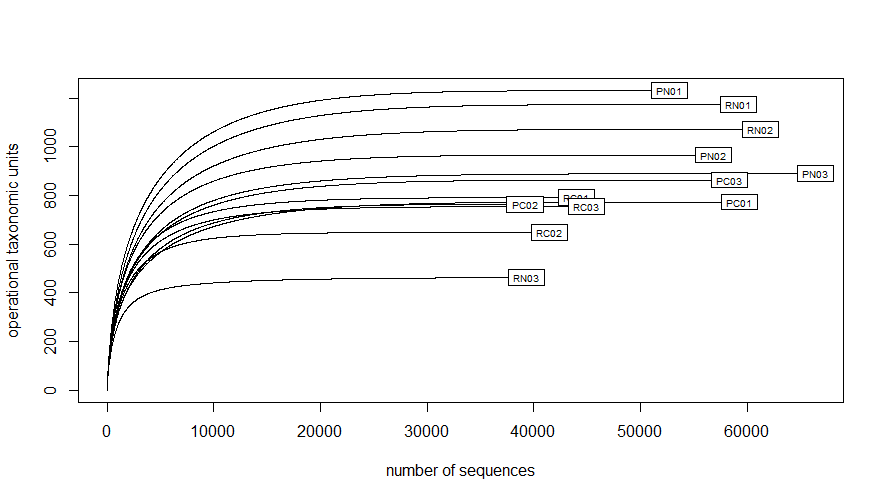

Supplement: S1 Fig — PN, Nantou polluted area; PC, Changhua polluted area; RN, Nantou remediated area; RC, Changhua remediated area. (TIFF) [file pone.0255137.s001.tiff]

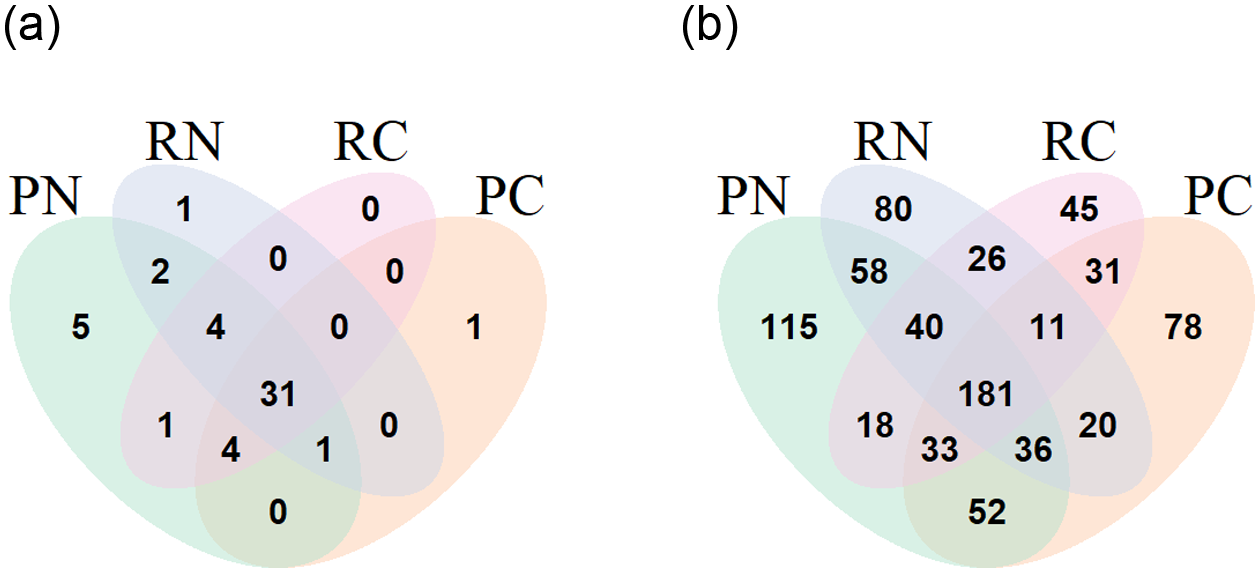

Supplement: S2 Fig — PN, Nantou polluted area; PC, Changhua polluted area; RN, Nantou remediated area; RC, Changhua remediated area. (TIF) [file pone.0255137.s002.tif]
